# Retrieval Practice and Word Learning by Children With Developmental Language Disorder: Does Expanding Retrieval Provide Additional Benefit?

**DOI:** 10.1044/2024_JSLHR-23-00528

**Published:** 2024-04-09

**Authors:** Laurence B. Leonard, Sharon L. Christ, Patricia Deevy, Jeffrey Karpicke, Justin B. Kueser

**Affiliations:** a Department of Speech, Language, and Hearing Sciences, Purdue University, West Lafayette, IN; b Department of Human Development and Family Science, Purdue University, West Lafayette, IN; c Department of Psychological Sciences, Purdue University, West Lafayette, IN

## Abstract

**Purpose:**

The word learning of preschool-age children with developmental language disorder (DLD) is improved when spaced retrieval practice is incorporated into the learning sessions. In this preregistered study, we compared two types of spacing—an expanding retrieval practice schedule and an equally spaced schedule—to determine if one of these approaches yields better word learning outcomes for the children.

**Method:**

Fourteen children with DLD aged 4–5 years and 14 same-age children with typical language development (TD) learned eight novel nouns over two sessions. Spacing for half of the novel words was expanded gradually during learning; for the remaining novel words, greater spacing remained at the same level throughout learning. Immediately after the second session and 1 week later, the children's recall of the words was tested.

**Results:**

The children with TD recalled more novel words than the children with DLD, although this difference could be accounted for by differences in the children's standardized receptive vocabulary test scores. The two groups were similar in their ability to retain the words over 1 week. Initially, the shorter spacing in the expanding schedule resulted in greater retrieval success than the corresponding (longer spaced) retrieval trials in the equally spaced schedule. These early shorter spaced trials also seemed to benefit retrieval of the trials with greater spacing that immediately followed. However, as the learning period progressed, the accuracy levels for the two conditions converged and were likewise similar during final testing.

**Conclusion:**

We need a greater understanding of how and when short spacing can be helpful to children's word learning, with the recognition that early gains might give a misleading picture of the benefits that short spacing can provide to longer term retention.

**Supplemental Material:**

https://doi.org/10.23641/asha.25537696

Many of us have benefited from our teachers' admonitions to spread our studying out over time instead of cramming all of our studying into a single period the night before the exam. However, this sage advice to distribute our studying is only part of the story. When we also test ourselves during our intermittent study periods, our retention is even better. It seems that testing is not simply an assessment of our learning; it is, itself, a form of learning.

This insight of the benefits of self-testing has long been part of the scientific literature, but in recent years, more has been learned about the process and the cognitive mechanisms that might be behind it. We continue those efforts in the present study by examining how testing during the learning period might assist the word learning of children with developmental language disorder (DLD). As in other studies, we use the term *retrieval* instead of *self-test* because children are explicitly asked to recall the material during the learning period. However, regardless of the term, the benefits are the same.

This study is the latest in a series of studies on retrieval effects, this time with the aim of testing the effects of alternative retrieval schedules on the word learning of children with DLD. We begin with a brief review of some of the principles of retrieval practice and follow with the evidence that led to the current study.

## The Benefits of Repeated Spaced Retrieval

Two consistent findings that undergird much of the basic research on retrieval are that (a) retrieval is more effective when it is repeated frequently (e.g., Roediger & Karpicke, 2006) and (b) retrieval is more successful when there is spacing between each retrieval attempt and the time when the material was last studied (Karpicke & Roediger, 2007). Often this spacing is created by inserting additional items between a study trial and the retrieval trial of the same item. Procedures that capitalize on both retrieval frequency and spacing are referred to as *repeated spaced retrieval* (RSR) procedures.

According to some scholars, one factor contributing to the facilitative effects of RSR is the temporal context of the learning event. Temporal context is “the current pattern of activity in an individual's mind . . . ” when an item is being studied (Bäuml, 2019, p. 177). When we recall an item, there is a partial reactivation of the context that was present during our initial study of the item. With successful retrieval of the item, this partial context combines with the present context to form a composite. Additional portions of context are incorporated in the composite with subsequent acts of retrieval, which renders the item increasingly distinct. With spacing, the contexts with each successive retrieval are less likely to be identical, which adds to the uniqueness of the composite that is gradually formed (Karpicke et al., 2014).

For tightly controlled experimental tasks, such as learning lists of items, differences in temporal context may seem minor given that items are often presented in a single session in a single location. However, even in such narrowly changing contexts, research participants succeed in remembering details such as the particular list an item appeared in even when they were never asked to pay attention to such details during the study period (Whiffen & Karpicke, 2017).

There is widespread evidence that RSR promotes learning relative to study alone (see meta-analysis by Rowland, 2014). Advantages for RSR have been found for the recall of a variety of materials—from learning English translations of Japanese words (Kang et al., 2014) to learning concepts in immunology (Dobson, 2012). Both a larger number of retrieval trials and greater spacing of these trials have been found to facilitate recall. These effects are even stronger when feedback is provided, that is, when the research participant has the opportunity to hear or see the item immediately after the retrieval attempt (Ma et al., 2020). Feedback provides the most benefit when the preceding retrieval attempt was incorrect or when it was correct but the participant had very little confidence in its accuracy (Butler et al., 2008; Rowland & DeLosh, 2015).

## RSR and Word Learning in Individuals With DLD

Because RSR provides a benefit to learning and recall of verbal materials, it has been applied in recent years to studies of individuals with DLD. These are individuals with a significant deficit in language ability of unknown origin (Bishop et al., 2017). Although weaknesses in these individuals can sometimes be found in areas beyond language itself, these other weaknesses do not represent causes of the language disorder. Genetic factors have been implicated in many cases of DLD, but the nature of the disorder appears to be multifactorial (Bishop et al., 2017). DLD is usually diagnosed during the later preschool or early elementary school years, but for many individuals, problems continue into adulthood (e.g., Dubois et al., 2020). As pointed out by McGregor (2020), children meeting the diagnostic criteria of DLD are significantly underserved, especially considering their prevalence of approximately 7.5% (Norbury et al., 2016; Tomblin et al., 1997) and the fact that they face many lifelong challenges of a communicative, academic, social, and even economic nature.

One of the language difficulties experienced by individuals with DLD is a significant weakness in word learning, seen in both children (e.g., McGregor et al., 2021; Storkel et al., 2017) and adolescents and adults (McGregor et al., 2017). Experimental studies indicate that individuals with DLD learn novel words more slowly and less accurately than their typically developing peers (e.g., Alt, 2011; Gray, 2003; Kan & Windsor, 2010). Most conspicuous is these individuals' difficulty with encoding—forming initial representations of new words (Bishop & Hsu, 2015; Gordon et al., 2021; Gray, 2004; Jackson et al., 2021). Longer term recall of adequately encoded words appears to be less impaired (McGregor et al., 2020).

Beginning in 2014, investigators began to examine whether the use of RSR might assist the word learning of individuals with DLD (e.g., Chen & Liu, 2014; McGregor et al., 2017). Results thus far have been encouraging (see Gordon, 2020, for a recent review).

In a series of studies, Leonard and colleagues examined the role of RSR in the word learning of 4- to 5-year-old children with DLD and their same-age peers with typical language development (TD; see reviews in Leonard & Deevy, 2020; Leonard et al., 2021). For example, in studies focused on words representing names of exotic plants and animals, the children were asked to learn novel word forms (e.g., /paɪb/) and the “meanings” assigned to the novel word forms (e.g., “a /paɪb/ likes butterflies”). Learning took place during two 20-min sessions held on consecutive days. In each session, the RSR conditions began with an immediate retrieval trial, defined as a request to recall the word form and meaning immediately after the child had just received the corresponding study trial (seeing the referent on a laptop screen and hearing the corresponding word form and meaning). Evenly spaced retrieval trials followed, with either two or three other words separating the retrieval trial from the last time the child received a study trial for the word to be retrieved, depending on the study. Five minutes after the second learning session and 1 week later, the children's recall of the word forms/meanings and their recognition of the word forms (e.g., “Where is the /paɪb/?”) were assessed.

Across studies, novel words learned in RSR conditions had better learning and recall outcomes than novel words assigned to comparison conditions that provided the same number of or even more exposures of each novel word. Comparison conditions included study-only conditions with no retrieval opportunities, immediate retrieval with no spacing, and more study opportunities but fewer retrieval opportunities (see below). Although all three outcome measures showed the RSR advantage when studied in the aggregate across studies (Leonard et al., 2021), the strongest and most consistent effects were seen for the recall of word forms. Specifically,

1.  novel words representing nouns (Leonard, Karpicke, et al., 2019), adjectives (Leonard, Deevy, et al., 2019), and verbs (Leonard et al., 2023) were learned and recalled more successfully through RSR than through repeated study alone;

2.  novel nouns were learned and recalled more successfully through RSR than through a schedule of repeated immediate retrieval with no spacing (Haebig et al., 2019);

3.  when the distribution of study trials and RSR trials was manipulated, learning and recall were greater when there were more retrieval trials and fewer study trials than when there were more study trials and fewer retrieval trials (Leonard et al., 2020);

4.  although immediate retrieval trials by themselves did not lead to greater recall, successful immediate retrieval trials early in the learning period led to more successful spaced retrieval trials during learning, which, in turn, was associated with greater recall after the learning period (Kueser et al., 2021);

5.  in some studies, the children with DLD recalled novel words to the same degree as same-age peers with TD, but when aggregated data across studies were examined, the typically developing peers showed greater learning and recall than the DLD groups (Leonard et al., 2021); and

6.  across studies, for the novel words that children with DLD managed to learn by the end of the learning period, their retention of the words over 1 week was no different from that of their peers when allowances were made for phonetic imprecision (Leonard et al., 2022).

Unfortunately, the clear relative advantages seen for the RSR condition in these studies did not translate into high absolute recall scores for either group of children. For example, only 63% of the novel noun word forms were recalled by the children with DLD at the end of the learning period (Leonard, Karpicke, et al., 2019); recall was worse for novel verb forms, at 35% (Leonard et al., 2023). Corresponding percentages for the children with TD were 79% and 46%, respectively. The RSR procedures, as we applied them, were far from ideal.

## Can an Expanding Retrieval Schedule Improve Word Learning and Recall?

An ever-present challenge in using spaced retrieval is finding the correct balance between selecting a degree of spacing that is large enough to promote long-term recall but not so large that it produces forgetting. Clearly, for many of our research participants, we did not find the right balance. Too many words showed no signs of being recalled.

In this preregistered study, we asked if an expanding spaced retrieval schedule would produce different outcomes than our more standard equally spaced retrieval schedule. In the most frequently used equally spaced retrieval schedule in the studies of Leonard and colleagues, an immediate retrieval trial (designated as a “0” trial because there were no intervening words) was then followed by evenly spaced retrieval trials with three other words separating the retrieval trial from the preceding study trial of the same word (designated as a “3” trial). In the present study, we compared an evenly spaced schedule of 03333 in each learning session with an expanding spaced schedule of 01133 in each session.

Expanding retrieval has been a well-researched method in the scientific literature on memory. The assumption behind this type of schedule is that, with gradual increases in the degree of spacing, early retrieval success is more likely and the small increments in spacing will keep forgetting to a minimum while retrieval effort increases. Early studies, including the influential work of Landauer and Bjork (1978), found that young adult participants recalled more items with expanding schedules (e.g., 1-4-10) than with equally spaced schedules (e.g., 5-5-5). Subsequent studies, however, have produced mixed results. Factors that apparently contribute to whether expanding retrieval is more effective than equally spaced retrieval include whether the first retrieval trial appears early (e.g., after one intervening item; Karpicke & Roediger, 2007), whether the item was successfully retrieved on a preceding trial (Karpicke & Bauernschmidt, 2011), whether the nature of intervening tasks had the potential to interfere with recall (Storm et al., 2010), whether feedback was provided (Karpicke & Roediger, 2007), whether recall success was measured over the course of the study or at final recall (Kang et al., 2014), and whether final recall was measured shortly after learning or several days later (Karpicke & Roediger, 2007). The participants' age, too, is a relevant factor; older adults require spacing increases in smaller increments than younger adults (Maddox et al., 2011).

Studies of expanding retrieval with children are limited in number. Fritz et al. (2007) found that preschool-age children with TD learned more new names of plush toys through an expanding retrieval schedule than with comparison conditions. However, a spaced retrieval condition with equal spacing was not among the conditions used for comparison.

Based on studies with young adult participants, we might expect an expanding retrieval schedule such as 01133 to show advantages over an equally spaced schedule such as 03333, but only during early trials. Karpicke and Roediger (2007) observed that previous findings of advantages of expanding retrieval over equally spaced retrieval were confounded by the fact that the previous studies included early retrieval trials (“0” and/or “1”) for the expanding retrieval condition, whereas all retrieval trials appeared later in the sequence in the equally spaced condition. Furthermore, when recall was tested days rather than minutes later, the advantage of expanding retrieval disappeared and, in some cases, equally spaced retrieval proved superior. In their own study, Karpicke and Roediger ensured that the timing of the first retrieval trial was the same across conditions and found that expanding retrieval was not superior. In fact, delaying the first retrieval trial produced better recall after 2 days, regardless of whether retrieval was expanding or equally spaced.

Karpicke and Roediger (2007) proposed that when retrieval trials occur shortly after study trials, the items have not cleared working memory and, therefore, the successful retrieval resulting from these early trials contributes relatively little to long-term retention. When retrieval trials are delayed, success is less assured, but the items that are retrieved at this point have more staying power.

Yet, findings from young adult participants may not apply to other groups. For example, Maddox et al. (2011) found that, for older adults (aged 65–89 years), spacing must be reduced relative to the spacing that is successful with young adults. Factors such as declining working memory capacity seem to be operating in older individuals. Note that this could mean that shorter spacing such as “1” might fall outside the bounds of a limited working memory capacity and may therefore function more like delayed spacing in younger adults. Because preschoolers' working memory capacity is likewise limited (and especially so in children with DLD; see, e.g., Jackson et al., 2020, 2021), it is possible that spacing that included “1” trials might represent the correct balance—reducing the likelihood of forgetting without relying primarily on working memory to retrieve the words.

A related reason for suggesting that expanding retrieval might be advantageous is that, in an earlier study on children with DLD, Kueser et al. (2021) found that immediate (“0”) retrieval trials, when successful, were associated with greater success on subsequent spaced (“3”) trials. Although success on immediate trials was not directly related to final recall accuracy, it may have served as a mediating factor—successful immediate retrieval trials increased the likelihood of successful spaced retrieval trials, which, in turn, increased the likelihood of success during final recall. Both conditions in the present study employed initial “0” trials, but it seemed possible that the inclusion of “1” trials in the expanding condition would further prepare the children for the greater spacing of “3” trials.

The present study followed the general design used in our previous studies. Preschoolers with DLD aged 4 and 5 years and their same-age peers with TD learned novel nouns over two sessions. Both word forms (e.g., /jʌt/) and meanings (e.g., “likes birds”) were taught. For each child, four of the novel words were taught in an expanding retrieval condition of 01133, and the remaining four novel words were taught in an equally spaced retrieval condition of 03333. Shortly after the second learning session, the children's recall of the word forms and meanings was assessed. One week later, the same recall tests were administered along with a recognition test.

Based on earlier findings, we expected the children with TD to show greater recall than the children with DLD, but the two groups would show very little decline from recall tested after the second session to recall tested 1 week later. The wholly new aspect of the present study is the comparison between two different schedules involving spaced retrieval. Based on all available evidence, it seemed that early shorter spaced “1” retrieval trials would be more successful than the corresponding longer spaced “3” trials of the 03333 condition. However, the results of the comparisons for longer term retention were more difficult to predict. Based on findings from young adult participants, the early advantage of short spacing would no longer hold for longer term recall. However, given the younger ages, language status, and more limited working memory capacities of our participants, it seemed highly plausible that expanding retrieval would continue to hold an advantage throughout the learning period and even during later testing.

Although we also examine meaning recall and novel word recognition, our emphasis is on the recall of word forms. Word form learning and recall have shown the greatest boost from RSR relative to comparison conditions. However, given that recall for word forms by children with DLD has remained low at an absolute level even with RSR (35%–63% depending on the study), this is the area of word learning in greatest need of more effective procedures. Here, we ask if an expanding retrieval (01133) schedule might prove to be one such type of improvement.

In our previous studies, scores from our other measures—meaning recall and recognition—have been much higher and have proved to be less informative. We included those measures in the present study to ensure that methods comparable to those in our previous studies were followed. The results for the postlearning meaning recall and recognition measures are presented in Supplemental Material S1.

## Method

This study was registered at clinicaltrials.gov (Laurence B. Leonard, NCT05325333), and all recruitment and experimental procedures were approved by the authors' institutional review board. Written consent was obtained from the children's families, and verbal assent was provided by the children.

### Participants

Twenty-eight children were participants in the study. All children who met the selection criteria were included, and no child left the study before testing had been completed.

#### Children With DLD

Fourteen of the children met the criteria for inclusion in the DLD group. Six girls and eight boys comprised the group with an *M*_age_ of 4;10 (years;months; *SD* = 6.67 months). These children were enrolled in language intervention programs or were scheduled to receive language intervention. These 14 children scored below 87 on the Structured Photographic Expressive Language Test–Preschool 2 (SPELT-P2; Dawson et al., 2005). This cutoff score has been found to show good sensitivity and specificity (Greenslade et al., 2009). All children scored above 75 (on the Kaufman Assessment Battery for Children, Second Edition [KABC-2]; Kaufman & Kaufman, 2004), a test of nonverbal intelligence, and scored in the “minimal to no symptoms” of autism spectrum disorder range on the Childhood Autism Rating Scale–Second Edition (CARS-2; Schopler et al., 2010). Each child passed a hearing screening in both ears at 20 dB at 500, 1000, 2000, and 4000 Hz.

Three additional measures were obtained that were not part of the selection criteria. As in our previous studies, two measures served as covariates—maternal education level measured in years of education and the children's standard scores on the Peabody Picture Vocabulary Test, Fifth Edition (PPVT-5; Dunn, 2019). The third measure was obtained for additional descriptive purposes—the Expressive Vocabulary Test, Third Edition (EVT-3; Williams, 2019) Because these three measures were not part of the selection criteria, the scores were free to vary. A summary of the children's scores on all measures can be seen in Table 1.

**Table 1.** Summary of the test scores and related information obtained from the children with developmental language disorder (DLD) and with typical language development (TD).

Variable	DLD (*n* = 14)	TD (*n* = 14)
Age in months	58.00 (6.67)	58.36 (7.02)
Sex	6 F, 8 M	7 F, 7 M
SPELT-P2 (SS)^a^	71.86 (12.10)	120.00 (5.33)
KABC-2 (SS)^a^	100.64 (17.81)	119.14 (10.30)
CARS-2^a^	16.43 (1.95)	—
PPVT-5 (SS)^b^	95.36 (13.89)	124.43 (12.49)
Maternal education in years^b^	16.21 (2.67)	16.57 (1.65)
EVT-3 (SS)^c^	93.86 (8.98)	119.07 (14.81)

*Note.* Em dashes indicate measure not administered. F = female; M = male; SPELT-P2 = Structured Photographic Expressive Language Test–Preschool 2; SS = standard score; KABC-2 = Kaufman Assessment Battery for Children, Second Edition; CARS-2 = Childhood Autism Rating Scale–Second Edition; PPVT-5 = The Peabody Picture Vocabulary Test, Fifth Edition; EVT-3 = Expressive Vocabulary Test, Third Edition.

a Selection criterion measure.

b Covariate measure.

c Additional clinical measure.

To anticipate any child's unusual productions of the novel words to be used in the study (see Novel Words section below), we constructed a production task of actual words that included the consonants and vowels contained in the novel words in the same word positions. For example, for the novel word /fun/, actual words on the production task included “food” and “fun”; for the novel word /gᴐf/, words included “good” and “off.” The actual words were presented in short phrases with the target word in final position that the child was asked to repeat (e.g., “Eat some food,” “This is fun,” “This is good,” “Turn that off!”). Our scoring procedures for the children's novel word productions allowed for some degree of phonetic imprecision (see Scoring and Reliability section below), but the actual-word production task gave us the possibility to observe instances of less expected productions such as, for example, labial assimilation (e.g., /bop/ or /pop/ for “soap”).

#### Children With TD

The 14 children with TD were similar to the children with DLD in age (*M* = 4;10, *SD* = 7.02 months). Seven children in the group were girls, and seven were boys. These children scored above 87 on the SPELT-P2 and above 75 on the KABC-2 and passed the hearing screening. The CARS-2 was not administered to these children as no developmental or educational concerns were reported by the children's parents.

The measures used for covariates (maternal education, PPVT-5 standard scores) and other descriptive purposes (EVT-3) were also obtained from the children in the TD group. Their scores also appear in Table 1. As was true for the children in the DLD group, the children in the TD group were also administered the speech production task involving actual words that contained the same consonants and vowels represented in the novel words used in the study.

### Novel Words

Eight novel words were presented to the children, four in each of two sets. The novel words were matched across sets according to biphone frequency and neighborhood density based on the child corpus-based values in Storkel (2013; see Supplemental Material S1). The novel words assigned to the same set differed in their initial consonant, vowel, and final consonant. The eight novel words—all consonant–vowel–consonant (CVC) monosyllables—were /nɛp/, /gᴐf/, /wæd/, /bog/, /jʌt/, /fun/, /paɪb/, and /dig/. The novel words served as the names of rare animals and exotic plants shown in color photographs on a laptop computer. The photographs were first used by McGregor (2014) and were also used in our subsequent studies that focused on novel nouns (Leonard et al., 2020; Leonard, Karpicke, et al., 2019).

### Procedure

The two sets of novel words were learned sequentially, with 1 week separating the end of testing for the first set and the beginning of the second set. For each set, the children participated in two 20-min learning sessions on consecutive days. At preplanned intervals during each session, the children were given the opportunity to choose a sticker and place it on a page (see Appendix A). During these very brief intervals, the children remained seated. The brief “sticker” breaks occurred regardless of the children's accuracy on the preceding retrieval item. At the end of the second session, the children were tested on their recall of the novel words, and 1 week later, they were tested on both their recall and recognition of the novel words (see below). One set of novel words appeared in an expanding spaced retrieval condition. In this condition, each of the two days employed a 01133 schedule for each word in the set. Appendix A provides an example of this schedule for the first day for one of the sets. The second day was identical to the first except the order of the novel words was reversed. The other set of novel words appeared in an equally spaced retrieval condition, with each word following a 03333 schedule each day. Appendix B shows an example for the first day for one of the sets. The order of the novel words was reversed for the second day. The order of the conditions and the words assigned to the conditions were counterbalanced across children within each participant group.

Each of the two learning sessions in each set began with two practice items consisting of actual words. Each practice item began with a study trial. For the first practice item, a photo of a rose was presented on the laptop, and the child heard the prerecorded description: “This is a rose. It's a rose. A rose likes water.” Note that, in this and in all other study trials, the word form (“rose” in this case) was heard three times and its “meaning” (that it likes water) was heard once. Following the study trial, an immediate retrieval trial occurred; the photo reappeared, and the child heard, “What's this called? What do we call this?” (a request for the word form) and “And what does this one like? What does it like?” (a request for the meaning). The second practice item followed the same study trial–immediate retrieval trial sequence. At that point, the novel words were presented.

For the novel words in both the expanding retrieval and equally spaced retrieval conditions, the session began with a study trial followed by an immediate retrieval (“0”) trial. Unlike the practice items, the “meaning” associated with each novel word form was arbitrarily assigned with the stipulation that nothing in the photo would suggest the meaning. An example is, “This is a yutt. It's a yutt. A yutt likes birds.” The immediate retrieval trial that followed the study trial had the same wording as the retrieval trial for the practice items (“What's this called? What do we call this?” and “What does this one like? What does it like?”), with a request for both the word form and its meaning. Following the immediate retrieval trial, the children received another study trial for the same novel word. This trial can be construed as feedback, although the children were not told if the preceding retrieval attempt was correct. All subsequent study trials that directly followed retrieval trials (see below) can likewise be viewed as feedback.

After each novel word in the set had a study trial–immediate retrieval trial–study trial sequence, the two conditions diverged. For words in the expanding retrieval (01133) condition, the next time the word appeared, it was in a retrieval trial after one other novel word from the same set had appeared (hence, a spaced “1” trial). A study trial occurred immediately after the retrieval trial. Another spaced “1” trial followed by a study trial then occurred.

After all words in the 01133 condition had been presented in two spaced “1” trial–study trial sequences, the children had a 2-min break, followed by an additional study trial for each novel word. This additional study trial for each word enabled us to ensure that all subsequent trials could be spaced “3” retrieval trials, in which three other words separated the retrieval trial and the last occasion the child heard the word in a study trial. Again, study trials followed these “3” trials. The learning session concluded with two more “3” trial–study trial sequences for each word. The same 01133 sequence was repeated on the second day.

For the words in the 03333 condition, after the initial “0” retrieval trial–study trial sequence, all words appeared in spaced “3” retrieval trials followed by a study trial. After all words had appeared in two spaced “3” retrieval trial–study trial sequences, the children had a 2-min break, after which an additional study trial occurred. Although this additional study trial was not needed to maintain the equal spacing of the retrieval trials, it matched the point in the sequence in which the additional study trial appeared in the 01133 sequence. Following the additional study trial, all words appeared in two more spaced “3” retrieval trial–study trial sequences. The second day was conducted in the same way as the first day.

The two conditions provided the children with the same number of presentations of each word form and meaning as well as the same number of retrieval opportunities. Specifically, in every study trial, the children heard the word form three times and the corresponding meaning once. With 14 study trials across the 2 days, there were a total of 42 presentations of each word form and 14 presentations of the meaning. There were 10 retrieval opportunities for each word form and its meaning.

### Postlearning Tests

Five minutes after completion of the second learning session of each set, the children were tested on their word form recall and meaning recall. The prompts used in this testing were the same prompts used for the retrieval trials during the learning period. Two items were used for each word form and meaning. After the four word forms and meanings were tested a first time, they were tested again, within the same list, resulting in eight test items per set.

One week later, the same word recall and meaning recall tests were administered, followed by a recognition test. Eight items were used in the recognition test. For each item, four photographs appeared on the laptop screen—the photograph matching the target word form and photographs matching the remaining words in the set. The prompt for each item was, “Which one is the (e.g., /dig/?) Where is the (e.g., /dig/)?” The child responded by pointing to the correct photograph. After the four words in the set were tested with one item each, they were tested again within the same list, although the location of the correct photo in the array was changed.

### Scoring and Reliability

Eight items were used for the word form recall test. Several steps were used in the scoring of the children's productions of the novel word forms. First, we ensured that the production was not an actual word that the child may have intended as an alternative name for the referent (e.g., “cactus”). These productions were scored as incorrect. Second, any productions that seemed to be potential attempts at the novel word were subjected to closer inspection. Many of the productions that deviated from adultlike pronunciation conformed closely to the children's pronunciations on the real-word production probe administered at the beginning of the study. As noted earlier, the real words on this pre-experiment probe (e.g., “food,” “fun”) contained consonants and vowels used in the novel words (e.g., /fun/). If a child had unusual substitutions for particular segments, this was taken into consideration. We then scored the candidate productions according to the scoring system of Edwards et al. (2004). This system was employed in our earlier studies and yielded excellent interscorer reliability. In the Edwards et al. system, each consonant is awarded 1 point each for correct place, manner, and voicing. For vowels, 1 point is given for each of length, height, and backness. An additional point is credited for correct syllable shape (CVC). Given that all novel words had the syllable shape CVC, all fully adultlike pronunciations earned 10 points. For any non-adultlike production to be scored as correct, the production was required to have a higher point total than the total that would be given if the child had instead been trying to produce one of the other novel words. For example, if a child produced the novel word /gᴐf/ as /gᴐp/, the production would be given 9 points (3 + 3 + 2 + 1). An alternative assumption that the child's production of /gᴐp/ was actually an attempt at /nɛp/ would lead to a score of 7 points (1 + 2 + 3 + 1). Given the lower score, this alternative interpretation (and others like it) would be rejected, and the production would be scored as correct recall of /gᴐf/.

The advantage of this method of scoring is that we could make judgments of correct/incorrect while still allowing for some phonetic imprecision on the children's part. Other methods of scoring have drawbacks. For example, simply using the entire range of scores (1–10) for each production without a discrete correct/incorrect decision could allow productions to be included in the data that were not actually intended as attempts at the novel word. Furthermore, requiring only fully adultlike productions would miss the fact that a child had consistently encoded a novel word somewhat incorrectly (as in /gᴐp/ for /gᴐf/) yet never applied that production when referring to another referent. With each score reflecting whether the item was deemed correct or incorrect, scores on the word form recall test ranged from 0 to 8.

Scoring of the children's responses on the meaning recall test was straightforward, as pronunciation, even when non-adultlike, was not a factor in distinguishing among the possible meanings (e.g., grass, butterflies, rain, sun). Scores could range from 0 to 8. The score for the recognition test was the number of items in which the child pointed to the correct photograph in the four-photograph array. With eight items, scores could range from 0 to 8.

To assess reliability for scoring the children's productions on the word form recall test, we selected the responses from four children in each group. A second judge independently scored these children's word form responses for both 5-min and 1-week testing of the novel words in both sets. Item-by-item interjudge reliability for correct–incorrect judgments using the Edwards et al. (2004) system was 96.88% for the DLD group and 100% for the TD group.

### Analysis Plan

The first set of analyses examined the children's longer term recall and recognition. Specifically, children's responses on the word form recall test, the meaning recall test, and the recognition test were evaluated using a series of mixed-effects models, with and without the covariates of PPVT-5 standard score and maternal education in years. The outcome was the number of correctly recalled items in a set of eight items (as two items were used for each of four novel words). Diagnostic group (DLD, TD) was a between-participant variable; within-participant variables were learning condition (01133, 03333) and time (5 min, 1 week; for the word form and meaning tests only). Random slopes for learning condition and time were included in the models when they were not close to zero. As a result, the random slope for the learning condition variable was included in the word form recall and meaning recall models, and the random slope for time was included in the meaning recall model.

Main effects models and full factorial models that included all possible two-way and three-way interactions were tested hierarchically. We present the main effects models with no interactions to provide baseline, pooled effects of each model variable. Only the meaning recall test had a statistically significant three-way interaction between group, condition, and time.

Effect sizes are reported as partially standardized beta coefficients (*b*_std_), which are comparable to a Cohen's *d* except they represent conditional standardized mean differences, conditioned on other variables in the model. Restricted maximum likelihood estimation was used. Bootstrapped standard errors with 1,000 replicates were used to account for nonnormal error terms in the meaning recall and recognition models. Stata (Version 17.0) was used for mixed-effects model analyses (StataCorp, 2019). The recall results for word form were of principal interest in this study. Results for meaning recall and recognition are presented in Supplemental Material S1.

The second set of analyses examined the children's trial-by-trial word form retrieval accuracy during the learning period. Quadratic trajectory models were run using mixed-effects logistic regression with PPVT-5 standard score, maternal education, and specific word to be retrieved serving as covariates. Models were run separately by group (DLD and TD) and day (1 and 2). Time in this case was the five retrieval points on each day (either 01133 or 03333). The linear change across time and the quadratic change across time were tested as random effects and included when they differed from zero. The linear time effect was included as a random effect only for the DLD group. Interactions of the linear and quadratic effects with spacing condition were included to allow for differences in change across condition.

Postmodel graphs of the trajectories by spacing condition were created using the model-based probability of correct retrieval. These graphs force a smoothed quadratic shape onto the data. Tests of differences in probability at each of the five retrieval points were evaluated using postmodel contrasts.

Predicted probabilities of retrieval at each trial were obtained from quadratic trajectory mixed-effects logit models that were the same as described above for trial-by-trial data but without the interactions. These probabilities were subsequently used as an independent variable in logistic regression models predicting correct retrieval of both items tested at 1 week (1 = *both items correct*, 0 = *no or one item correct*). Very few cases (3.12%) of children correctly retrieved one out of two items. These models were full-factorial models with the trial probabilities, time point, day, and group as independent variables. Postmodel graphs of the probability of correct retrieval of both tests at 1 week as a function of trial (retrieval time point) probabilities by spacing condition, group, and day were created.

## Results

### Postlearning Word Form Recall

Word form recall differences between the two learning conditions were minimal. As can be seen from Table 2, word form recall scores for the expanding retrieval condition (01133) were 0.59 points higher (on a 0–8 scale) than scores for the equally spaced condition (03333). The small-to-medium effect size observed (*b*_std_ = 0.23) was not different from zero. Likewise, we found no discernable difference between word form scores at 5 min and scores at 1 week (*b*_std_ = −0.05).

**Table 2.** Main effects word form model results (*N* = 28, *o* = 112).

Fixed effects	Main effects: no covariates					Main effects: with covariates				
	*b*	95% CI		*b*_std_	*p* value	*b*	95% CI		*b*_std_	*p* value
Group (DLD vs. TD)	−1.88	−3.70	−0.07	−0.72	.042	0.79	−1.68	3.26	0.30	.531
Condition (01133 vs. 03333)	0.59	−0.42	1.60	0.23	.252	0.59	−0.42	1.60	0.23	.252
Time (1 week vs. 5 min)	−0.13	−0.47	0.22	−0.05	.475	−0.13	−0.47	0.22	−0.05	.474
Covariates										
PPVT						0.09	0.03	0.16	0.03	.006
Mother's education						0.11	−0.27	0.49	0.04	.574
Intercept	5.47	4.17	6.77		.000	−7.64	−17.25	1.98	−4.74	.120
**Random effects**	**σ** ^**2**^	**95% CI**				**σ** ^**2**^	**95% CI**			
Condition	6.56	3.57	12.07			6.56	3.57	12.07		
Intercept	5.62	3.12	10.12			4.42	2.37	8.25		
Residual	0.86	0.58	1.27			0.86	0.58	1.26		

*Note.* DLD = developmental language disorder; TD = typical language development; PPVT = Peabody Picture Vocabulary Test.

**Figure 1.**
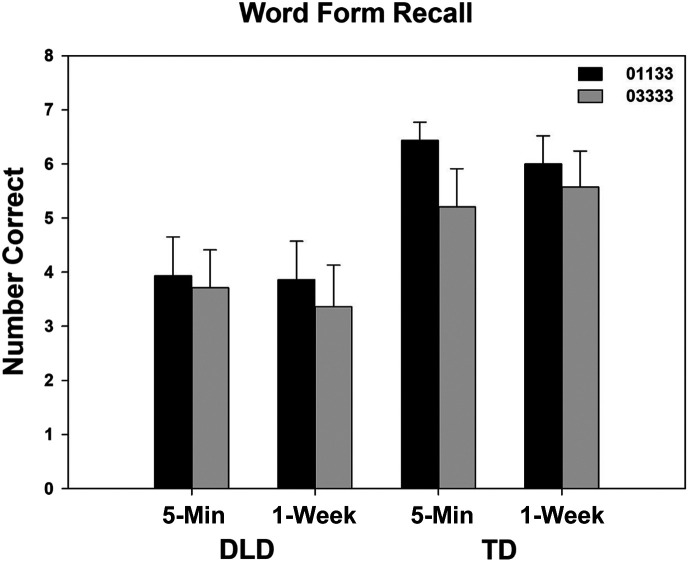
Mean recall scores for the children with developmental language disorder (DLD) and the children with typical language development (TD) at 5-min and 1-week testing. Novel words appeared in an expanding recall (01133) or equally spaced recall (03333) condition. Error bars indicate standard errors.

Differences according to participant group were influenced by the application of the covariates. The children with TD had numerically higher word form recall scores than the children with DLD. However, when the covariates were applied, the effect size was reduced (*b*_std_ = 0.30) and no longer different from zero. That is, although TD scores were higher, the measures used as covariates could account for these differences. As seen in Table 2, PPVT-5 scores, in particular, were likely playing a larger role than maternal education in this regard. It can be noted from Table 1 that the children with TD had much higher PPVT-5 scores than the children with DLD, whereas the mothers of the children in the two groups were quite similar in years of education. No interactions were observed. An illustration of the results can be seen in Figure 1.

### Trial-by-Trial Word Form Retrieval Accuracy

Previous studies with young adult participants have shown that although expanding retrieval may not show an advantage over equally spaced retrieval when measured over the long term, early short-spaced trials do offer an advantage over early larger spaced trials during the initial phase of learning. To determine whether this was true for our participants, we ran quadratic trajectory models using mixed-effects logistic regression of the trial-by-trial data. Because the trials spanned 2 days and repeated the same sequence on the second day (either 01133 or 03333), models were run separately for each day.

**Figure 2.**
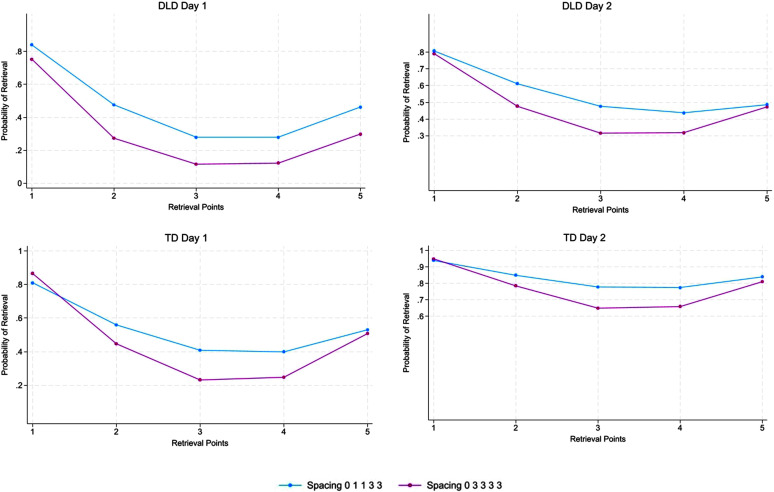
Probability of retrieval at each retrieval point during the learning period. Retrieval Points 1–5 correspond to 01133 spacing in the expanding retrieval condition and to 03333 in the equally spaced retrieval condition. DLD = developmental language disorder; TD = typical language development.

Figure 2 shows the children's probability of retrieving a novel word at each time point by group (DLD, TD) and by day (1, 2). For the children with DLD on Day 1, there was no difference in linear (*p* = .332) or quadratic (*p* = .361) change between the expanding and equally spaced learning conditions. Table 3 provides the probabilities at each time point. As expected, the shorter “1” retrieval trials of the 01133 condition (Points 2 and 3) had higher probabilities than the corresponding “3” trials of the 03333 condition. The higher probabilities for 01133 continued at points 4 and 5, although these were “3” trials for both conditions. On Day 2 for the DLD group, there was similarly no difference in either the linear (*p* = .142) or quadratic (*p* = .123) change between the 01133 and 0333 conditions. As was seen for Day 1, the probabilities were higher for the 01133 condition with the important exception of the final trial, where the probabilities for the two conditions converged, no longer showing a difference. By the final trial, then, retrieval success was no different between the expanding and equally spaced retrieval learning conditions, much as we found for the 5-min and 1-week word form recall tests that were administered after the learning period.

**Table 3.** The probability of successfully retrieving a novel word at each retrieval point during the learning period, by group, day, and spacing condition.

Retrieval point	1	2	3	4	5
Spacing	*0*	*1*	*1*	*3*	*3*
	*0*	*3*	*3*	*3*	*3*
DLD Day 1					
Spacing 01133 probability	.841	.476	.279	.279	.462
Spacing 03333 probability	.752	.274	.116	.123	.298
*p* value on difference	.137	.000	.000	.000	.026
DLD Day 2					
Spacing 01133 probability	.809	.612	.476	.436	.487
Spacing 03333 probability	.791	.477	.315	.317	.472
*p* value on difference	.632	.006	.003	.007	.679
TD Day 1					
Spacing 01133 probability	.809	.559	.409	.400	.531
Spacing 03333 probability	.867	.447	.232	.248	.509
*p* value on difference	.286	.047	.001	.002	.824
TD Day 2					
Spacing 01133 probability	.941	.851	.778	.774	.841
Spacing 03333 probability	.949	.787	.650	.660	.811
*p* value on difference	.546	.262	.048	.065	.874

*Note.* The retrieval points correspond to the spacing levels of each condition, with the first retrieval point corresponding to the “0” in both conditions and the fifth retrieval point corresponding to the final “3” in both conditions. The two conditions differed in spacing at the second and third retrieval points. The *p* values are for tests of differences between the two conditions at each retrieval point. DLD = developmental language disorder; TD = typical language development.

The findings were somewhat different for the children with TD. There were differences in linear (*p* = .012) and quadratic (*p* = .014) change between the expanding and equally spaced conditions on Day 1. Probabilities of retrieval were higher for the 01133 condition at Points 2 and 3 when “1” trials were compared to “3” trials and also at Point 4 when “3” trials appeared in both conditions. However, the probabilities for the two conditions converged at the final point for Day 1, in contrast to what we found for the children with DLD at the end of Day 1.

The retrieval probabilities for the TD group on Day 2 showed no difference in linear (*p* = .121) or quadratic (*p* = .127) change between the two learning conditions. Given that retrieval probability differences were no longer apparent at the end of Day 1 for the TD group, we might have expected the two conditions to show very similar probabilities throughout the Day 2 learning period. This was generally true; however, as seen in Table 3, we did find a difference at Retrieval Point 3 involving a comparison between a “1” trial and a “3” trial. Thereafter, differences were no longer seen.

### How Well Does Retrieval Success During Learning Predict Final Recall?

An assumption behind the use of greater spacing is that early attempts at retrieval may not be successful but, as retrieval gradually becomes more successful, longer term retention is more likely. We examined this basic assumption by comparing key retrieval points in the two conditions to determine the degree to which successful retrieval at these points predicted final recall accuracy. Of particular interest were the third retrieval point that involved different degrees of spacing in the two conditions (“1” vs. “3”) and the fourth retrieval point that had the same degree of spacing (“3”) in the two conditions. As we saw in Table 3, at both of these retrieval points, retrieval was more successful in the 01133 condition for the children with DLD on both days and for the children with TD on the first day.

**Figure 3.**
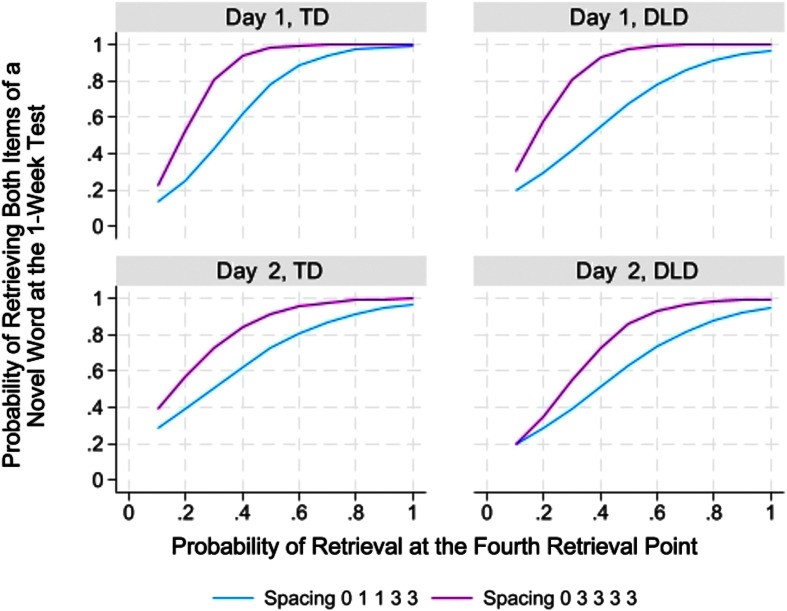
Probability of correct recall of both items of a novel word 1 week after the learning period as a function of the probability level of retrieving the word at the fourth retrieval point, which had “3” spacing in each condition. DLD = developmental language disorder; TD = typical language development.

Yet, the higher success rates in the 01133 condition during learning did not translate into better final recall. In Figure 3, we show an example for Retrieval Point 4 (corresponding to a “3” trial in both conditions). The figure provides the probability of correct recall at 1 week as a function of the probability of correct recall of a word at Retrieval Point 4 during the learning period. As can be seen, at no point is the probability higher for the 01133 condition. In fact, when children retrieved an item with .40 probability during Retrieval Point 4 on the first day of learning, the words in the 03333 condition were actually more likely to be correct at final recall than the words in the 01133 condition. As can be seen in Table 4, this was true for both groups of children and true as well for Retrieval Point 3 where the 01133 condition required retrieval with “1” spacing. Figure 3 also shows that as the probability of retrieving a word increased further, the difference favoring the 03333 condition began to wane. Perhaps not surprisingly, when a word had a very high probability of being retrieved at Retrieval Point 4, the word was also highly likely to be recalled 1 week later, regardless of the condition in which the word appeared.

**Table 4.** Comparisons between the expanding (01133) and equally spaced (03333) retrieval conditions for a probability of .40 at Retrieval Points 3 and 4.

Retrieval point	Difference in probability at 1 week	*SE*	*p* value	95% CI	
Time point 3					
TD Day 1	0.37	0.099	.001	0.16	0.57
TD Day 2	0.24	0.158	.139	−0.08	0.57
Time point 3					
DLD Day 1	0.40	0.131	.005	0.13	0.67
DLD Day 2	0.28	0.163	.093	−0.05	0.62
Time point 4					
TD Day 1	0.33	0.095	.002	0.13	0.52
TD Day 2	0.22	0.156	.175	−0.10	0.54
Time point 4					
DLD Day 1	0.38	0.143	.013	0.08	0.67
DLD Day 2	0.22	0.166	.203	−0.12	0.56

*Note.* All differences favored the equally spaced condition. TD = typical language development; DLD = developmental language disorder.

## Discussion

### Postlearning Word Form Recall

Two of the comparisons made in the postlearning word form recall analysis matched those in our prior studies—comparisons between DLD and TD groups and comparisons between 5-min and 1-week testing. In our earlier work, both the DLD and TD groups benefited from RSR, but in the aggregate, scores were higher overall for the TD group even when covariates were applied (Leonard et al., 2021). The exception was seen in the most recent study on novel verb learning (Leonard et al., 2023). In that study, the group with TD clearly recalled more word forms than the children with DLD. However, when the standard scores from the PPVT-4 were applied as a covariate, the PPVT-4 differences statistically accounted for the group difference in word form recall. We speculated that this exception to our previous findings was due to our use of novel verbs rather than novel nouns or adjectives.

However, the covariate also played a role in the present study, although novel nouns were employed. The children's initial PPVT-5 scores could account for what were otherwise clear differences between the two groups' word form recall. In this latest study, the newest standardization of the PPVT was used (PPVT-5 instead of PPVT-4), but it is unclear if this had a bearing on the results. We certainly do not conclude from this finding that the children with DLD were similar to the children with TD in their word form learning and recall. They were clearly less successful than their typically developing peers in this regard. Rather, we were unable to demonstrate that these differences were independent of the type of knowledge that underlies performance on the PPVT-5.

As noted earlier, although not a main focus of our study, a recognition task (e.g., “Where's the /dik/?”) was also administered to the children. Scores on this task were higher than those on the word form recall task for both groups (see Figure B in Supplemental Material S1). However, just as we saw for recall, a clear difference favoring the TD group could be statistically accounted for by the group differences in PPVT-5 scores. In this case, both measures (recognition task, PPVT-5) were receptive, picture-pointing tasks.

The ability of the PPVT-5 to account for group differences might have been inflated given our scoring criteria. Recall that we scored as correct those productions that subjectively appeared to be attempts at the correct novel word and whose phonetic details were closer to the correct novel word than they were to any of the other novel words based on the system of Edwards et al. (2004). These criteria left room for phonetic imprecision (e.g., /gᴐp/ instead of /gᴐf/). It is not clear that any differences between the groups in these finer phonetic details could have been accounted for by the PPVT-5.

The second comparison shared with our earlier studies was the comparison between word form recall at 5 min and 1 week. As in our earlier work, we found no decline in recall over time, and no interactions were seen. It appears that longer term retention, at least as defined as over 1-week time, does not seem to be a major factor in the word form learning challenges faced by children with DLD.

We believe that the similar 1-week stability in the two groups coupled with the overall lower scores for the DLD group implicates encoding as the principal weakness in the children with DLD. These children managed to adequately encode fewer words than their peers by 5-min testing. However, for those words that were adequately encoded, their recall 1 week later was indistinguishable from that of their peers. These findings accord with the findings of previous studies of our own and of other laboratories (e.g., Gray, 2004; McGregor et al., 2017).

We were especially interested in the remaining comparison, between an expanding retrieval schedule and an equally spaced retrieval schedule. This is a new type of comparison—pitting two different RSR schedules against one another in a word learning study involving children. However, we found no differences between the two schedules during postlearning recall testing. Neither group at either 5 min or 1 week had word form recall scores approaching ceiling levels (and there were no floor effects); therefore, there was an opportunity for differences to emerge if they were present.

The postlearning results were in line with findings from studies with young adult participants; the inclusion of early short-spaced retrieval trials did not provide any special benefits to longer term retention. The fact that the same proved true for this study was disappointing, especially because our usual RSR schedules had not produced high word form recall scores on an absolute basis. They were advantageous only relative to other non-RSR learning conditions.

### Trial-by-Trial Condition Effects During Learning

There were two possible reasons why the expanding 01133 schedule was not more effective in the end than our equally spaced 03333 schedule. The first is that the shorter spaced “1” trials were no easier for the children than the larger spaced “3” trials. However, this does not appear to be true. As we saw in Table 3, the “1” trials were retrieved with greater success than the corresponding “3” trials in the 03333 condition for both groups of children. The second possible reason is that the shorter spaced “1” trials were easier but provided no special benefit for retrieval at later points when larger spaced “3” trials were used. At first blush, this second reason also seems incorrect because the “3” trials most closely following the “1” trials in the 01133 condition showed higher probabilities of successful retrieval than the corresponding “3” trials in the 03333 condition. However, by later trials, the beneficial effects of “1” trials weakened and were no longer apparent in the “3” trials appearing at the end of the first day and thereafter for the TD group and in the “3” trials at the end of the second day for the DLD group.

The finding that retrieval during “3” trials seemingly benefited from immediately preceding “1” trials does not square with an assumption that the “1” trials fell within the boundaries of the children's working memory spans and therefore provided the children with a qualitatively different means of successful retrieval than was necessary for success with greater spacing. If the memory systems were different for “1” and “3” trials, we might have expected a marked decline in retrieval probability once the “3” trials were initiated in the 01133 condition. As can be seen in Figure 2, declines were minimal and, in fact, smaller than the declines from the first to the second “1” trial.

### Do Shorter and Longer Spacing Make Different Contributions to Learning?

How is it possible that “1” trials assisted retrieval of novel word forms in the “3” trials that immediately followed yet, by the end of the learning period, provided no greater benefit than the 03333 condition that never included “1” trials? We could certainly understand the possibility that children's feelings of success on a “1” trial could lead to their willingness to engage in the more effortful retrieval required on a subsequent “3” trial. However, even if this willingness led to earlier success on the “3” trial than would otherwise have been the case, this interpretation provides no explanation for why success on “3” trials in the 03333 condition eventually reached the same level.

Another possibility is that there were two offsetting factors. The shorter spacing may have facilitated the children's initial encoding of the novel word form, which, in turn, made subsequent retrieval in the short term become more likely. However, this early advantage was gradually erased due to differences in the contributions of changes in temporal context. With “3” spacing for all four spaced retrieval trials, the 03333 condition provided more opportunities for the temporal context to change with each retrieval attempt. According to this type of account (Karpicke et al., 2014), repeated retrieval in contexts that have changed can lead to the gradual building of a more distinct composite, which promotes greater long-term retention. In short, the 03333 condition was more difficult early in the process by virtue of greater spacing, but with a gradual buildup of a more distinctive composite, much of the early disadvantage was erased.

Consistent with this interpretation is our finding that although retrieval of words with “3” spacing at the fourth retrieval point was more successful in the 01133 condition than in the 03333 condition, the words that were successfully retrieved in the 03333 condition at that point were nevertheless just as likely, if not more likely, to be retained at final recall. The fact that the fourth retrieval point represented the third “3” trial for the word in the 03333 condition rather than only the first as in the 01133 condition might have enabled retrieved words in the 03333 condition to be more accessible, thanks to their more distinctive composites.

## Conclusions

Given the intuitive appeal of increasing spacing gradually for young learners with language deficits, it would be natural to propose a future study that employs a different expanding retrieval schedule. For example, a 01223 schedule might provide an even smoother transition into the more challenging “3” spacing than the 01133 schedule that we employed.

However, a greater understanding of spacing effects will likely require closer examination of why shorter spacing can provide temporary—but only temporary—advantages for retrieval with greater spacing. As we have speculated, there might be a trade-off between encoding and temporal context composite uniqueness. Shorter spacing might assist the children's initial word form encoding. However, repeated retrieval with greater spacing might eventually make up the difference, thanks to the more distinctive composite that can be formed with multiple acts of retrieval in changing contexts. By understanding the specific contributions made by different degrees of spacing, we might arrive at more effective ways of applying retrieval practice to promote greater word learning in children.

## Data Availability Statement

The data sets used for this study are available from the corresponding author on reasonable request.

## Supplementary Material

10.1044/2024_JSLHR-23-00528SMS1 Supplemental Material S1 Post-learning meaning recall and post-learning recognition.

## Appendix A

### Schedule for Condition 01133 on Day 1

**Table:**

Trial	Word form	Meaning (it likes . . . )	Spacing	Trial sequence
practice	Rose	water	0	study-retrieval
practice	Moose	carrots	0	study-retrieval
READY?				
1	/nɛp/	grass	0	study-retrieval-study
2	/gɔf/	sun	0	study-retrieval-study
3	/nɛp/	grass	1	retrieval-study
4	/gɔf/	sun	1	retrieval-study
5	/nɛp/	grass	1	retrieval-study
6	/gɔf/	sun	1	retrieval-study
*Sticker break*				
7	/wæd/	butterflies	0	study-retrieval-study
8	/bog/	rain	0	study-retrieval-study
9	/wæd/	butterflies	1	retrieval-study
10	/bog/	rain	1	retrieval-study
11	/wæd/	butterflies	1	retrieval-study
12	/bog/	rain	1	retrieval-study
*Sticker break*				
13	/nɛp/	grass	—	study
14	/gɔf/	sun	—	study
15	/wæd/	butterflies	—	study
16	/bog/	rain	—	study
17	/nɛp/	grass	3	retrieval-study
18	/gɔf/	sun	3	retrieval-study
19	/wæd/	butterflies	3	retrieval-study
20	/bog/	rain	3	retrieval-study
*Sticker break*				
21	/nɛp/	grass	3	retrieval-study
22	/gɔf/	sun	3	retrieval-study
23	/wæd/	butterflies	3	retrieval-study
24	/bog/	rain	3	retrieval-study

## Appendix B

### Schedule for Condition 03333 on Day 1

**Table:**

Trial	Word form	Meaning (it likes . . . )	Spacing	Trial sequence
practice	Rose	water	0	study-retrieval
practice	Moose	carrots	0	study-retrieval
READY?				
1	/jʌt/	birds	0	study-retrieval-study
2	/fun/	snow	0	study-retrieval-study
3	/pɑɪb/	wind	0	study-retrieval-study
4	/dig/	clouds	0	study-retrieval-study
5	/jʌt/	birds	3	retrieval-study
6	/fun/	snow	3	retrieval-study
7	/pɑɪb/	wind	3	retrieval-study
8	/dig/	clouds	3	retrieval-study
*Sticker break*				
9	/jʌt/	birds	3	retrieval-study
10	/fun/	snow	3	retrieval-study
11	/pɑɪb/	wind	3	retrieval-study
12	/dig/	clouds	3	retrieval-study
*Sticker break*				
13	/jʌt/	birds	—	study
14	/fun/	snow	—	study
15	/pɑɪb/	wind	—	study
16	/dig/	clouds	—	study
17	/jʌt/	birds	3	retrieval-study
18	/fun/	snow	3	retrieval-study
19	/pɑɪb/	wind	3	retrieval-study
20	/dig/	clouds	3	retrieval-study
*Sticker break*				
21	/jʌt/	birds	3	retrieval-study
22	/fun/	snow	3	retrieval-study
23	/pɑɪb/	wind	3	retrieval-study
24	/dig/	clouds	3	retrieval-study
