# Supplementary material for: Retrieval Practice and Word Learning by Children With Developmental Language Disorder: Does Expanding Retrieval Provide Additional Benefit?
Source: J Speech Lang Hear Res. 2024 Apr 9;67(5):1530–47. doi: 10.1044/2024_JSLHR-23-00528 (PMC11087082; doi:10.1044/2024_JSLHR-23-00528)
Supplement: Supplemental Material S1 [file JSLHR-67-1530-s001.pdf]

https://doi.org/10.1044/2024_JSLHR-23-00528

Supplemental Material S1. Post-learning meaning recall and post-learning recognition.

Post-Learning Meaning Recall

We found no main effect associated with learning condition for meaning recall, as shown in Supplemental Table A. Recall scores were generally quite high. The small effect (b_{std} = 0.11) was not different from zero. A small effect for participant group (b_{std} = -0.16) was similarly statistically unreliable. The covariates played no role in the participant group comparison, unlike our finding for word form recall.

Supplemental Table A. Main effects meaning model results (*N* = 28, *o* = 112).[a]

	**Main effects - no covariates**					**Main effects - with covariates**				
Fixed effects	*b*	95% CI		b_{std}	*p*-value	*B*	95% CI		b_{std}	*p*-value
Group (DLD vs TD)	-0.23	-1.32	0.85	-0.16	.672	-0.28	-1.50	0.93	-0.20	.647
Condition (01133 vs 03333)	0.20	-0.42	0.81	0.11	.534	0.20	-0.40	0.79	0.11	.515
Time (1wk vs 5min)	-0.38	-0.58	-0.17	-0.21	.000	-0.38	-0.58	-0.17	-0.21	.000
Covariates										
PPVT						0.00	-0.03	0.03	0.00	.955
Mother's Education						-0.08	-0.30	0.14	-0.05	.495
Intercept	7.41	6.63	8.20		.000	8.78	4.81	12.75		.000
Random effects	$σ^2$					$σ^2$				
Condition	3.98	1.66	9.56			4.01	1.76	9.12		
Time	0.19	0.10	0.36			0.18	0.09	0.36		
Intercept	3.42	0.47	24.75			3.70	0.55	25.03		
Residual	0.38	0.17	0.85			0.38	0.17	0.83		

[a]Bootstrap standard errors were used.

Differences were apparent for time. Meaning recall scores were 0.38 points (on a scale from 0 to 8) higher at five minutes than at one week, indicating a decline (b_{std} = -0.21) across time that was different from zero (p < .001). However, this effect is best interpreted with reference to a three-way interaction of group by learning condition by time (b_{std} = 0.61, p = .014). The simple effects of this three-way interaction are shown in Supplemental Table B. The driving force behind the interaction was the major decline in scores across time in the equally spaced (03333) condition for the children with DLD (b_{std} = -0.44) that was not seen for the children with TD (b_{std} = 0.04). The meaning recall results are illustrated in Supplemental Figure A.

Supplemental Table B. Simple effects for the group by condition by item interaction.[a]

	b	95% CI		b_{std}	*p*-value
DLD versus TD for 03333 condition at 5 minutes	0.10	-1.23	1.42	0.05	.887
DLD versus TD for 01133 condition at 5 minutes	-0.47	-1.60	0.65	-0.27	.410
DLD versus TD for 03333 condition at 1 week	-0.76	-2.13	0.61	-0.43	.278
DLD versus TD for 01133 condition at 1 week	-0.26	-1.63	1.11	-0.15	.710
01133 versus 03333 for TD group at 5 minutes	0.50	-0.45	1.45	0.28	.304
01133 versus 03333 for DLD group at 5 minutes	-0.07	-1.12	0.97	-0.04	.893
01133 versus 03333 for TD group at 1 week	-0.07	-0.75	0.60	-0.04	.836
01133 versus 03333 for DLD group at 1 week	0.43	-0.66	1.52	0.24	.442
1 week versus 5 minutes for TD group and 03333 condition	0.07	-0.20	0.34	0.04	.602
1 week versus 5 minutes for DLD group and 03333 condition	-0.79	-1.28	-0.29	-0.44	.002
1 week versus 5 minutes for TD group and 01133 condition	-0.50	-1.06	0.06	-0.28	.079
1 week versus 5 minutes for DLD group and 01133 condition	-0.29	-0.57	0.00	-0.16	.053

[a]Bootstrapped standard errors.

Supplemental Figure A. Meaning recall results.

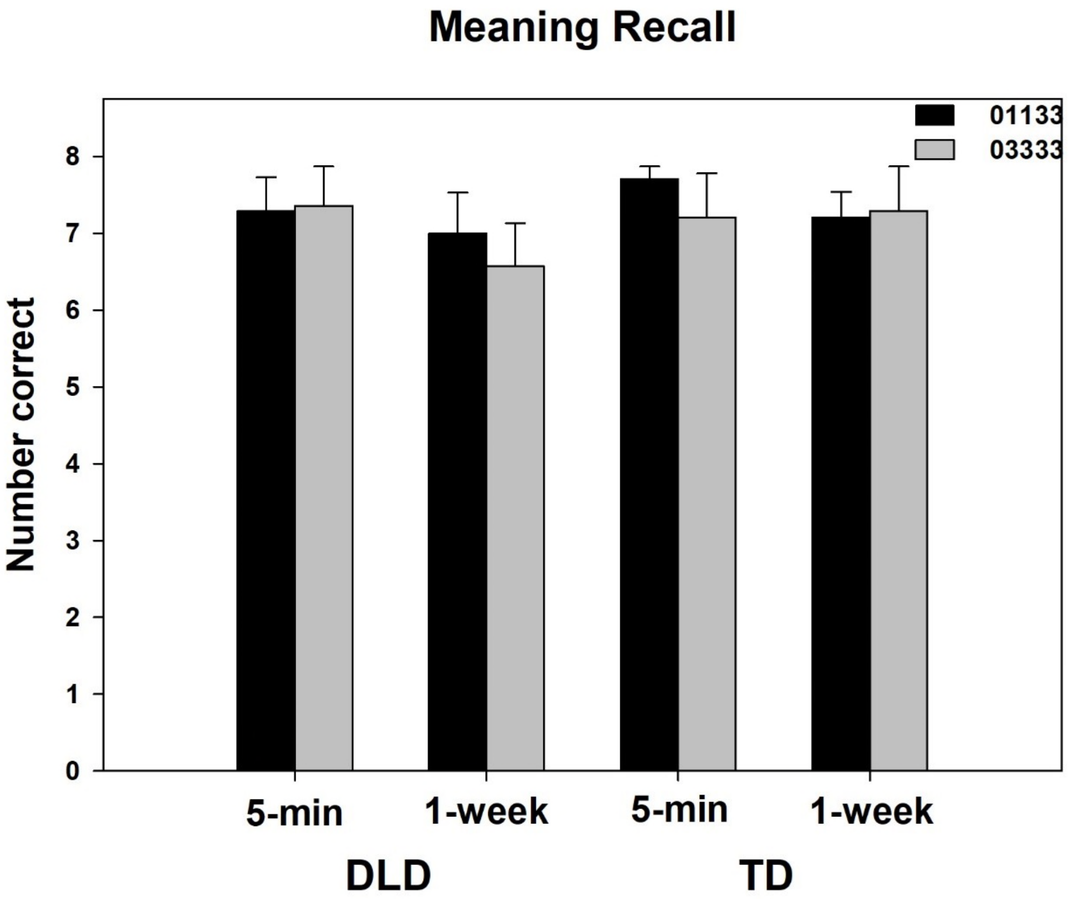

It is tempting to interpret the interaction as a sign that the 01133 condition allowed the children with DLD to hold on to meanings longer than was true for the 03333 condition. However, in previous studies of meaning recall, equally spaced schedules similar to our 03333 condition were not associated with a decline over time in children with DLD. The types of meanings to be learned—what unusual plants and animals “liked”—were also the same across these studies. Therefore, along with the possibility that the expanding 01133 schedule protected against forgetting over time, there is the possibility that there was some undetermined factor that made the equally spaced 03333 schedule in the present study less conducive to longer-term recall for meanings.

Post-Learning Recognition

The recognition task was administered only at the one week point and therefore analyses focused on learning condition and participant group. Results are shown in Supplemental Table C and illustrated in Supplemental Figure B. Recognition scores were 0.54 points higher (on a 0 to 8 scale) for words in the 01133 condition than for words in the 03333 condition. However, the medium effect size observed (b_{std} = 0.43) was not different from zero. As seen in Supplemental Figure B, scores were generally high with many children at ceiling. This fact may have made it difficult to discover differences between the learning conditions. Of the three measures used in our previous studies—word form recall, meaning recall, and recognition—the recognition test has routinely produced the smallest effect sizes (Leonard et al., 2021) due to high scores from many of the children.

Supplemental Table C. Main effects recognition model results (*N* = 28, o = 56).[a]

	**Main effects - no covariates**					**Main effects - with covariates**				
Fixed effects	*b*	95% CI		b_{std}	*p*-value	*b*	95% CI		b_{std}	*p*-value
Group (DLD vs TD)	-0.96	-1.48	-0.44	-0.78	.000	-0.36	-1.27	0.55	-0.288	.442
Condition (01133 vs 03333)	0.54	-0.02	1.09	0.43	.058	0.54	-0.01	1.08	0.431	.053
<u>Covariates</u>										
PPVT						0.02	0.00	0.04	0.018	.046
Mother's Education						-0.08	-0.25	0.09	-0.062	.378
Intercept	7.59	7.25	7.93		.000	6.15	2.41	9.88		.001
Random effects	σ^2					σ^2				
Intercept	0.16	0.00	71.42			0.11	0.00	2089.05		
Residual	1.13	0.43	2.94			1.13	0.43	3.00		

[a]Bootstrap standard errors were used.

Supplemental Figure B. Main effects recognition model results (*N* = 28, *o* = 56).

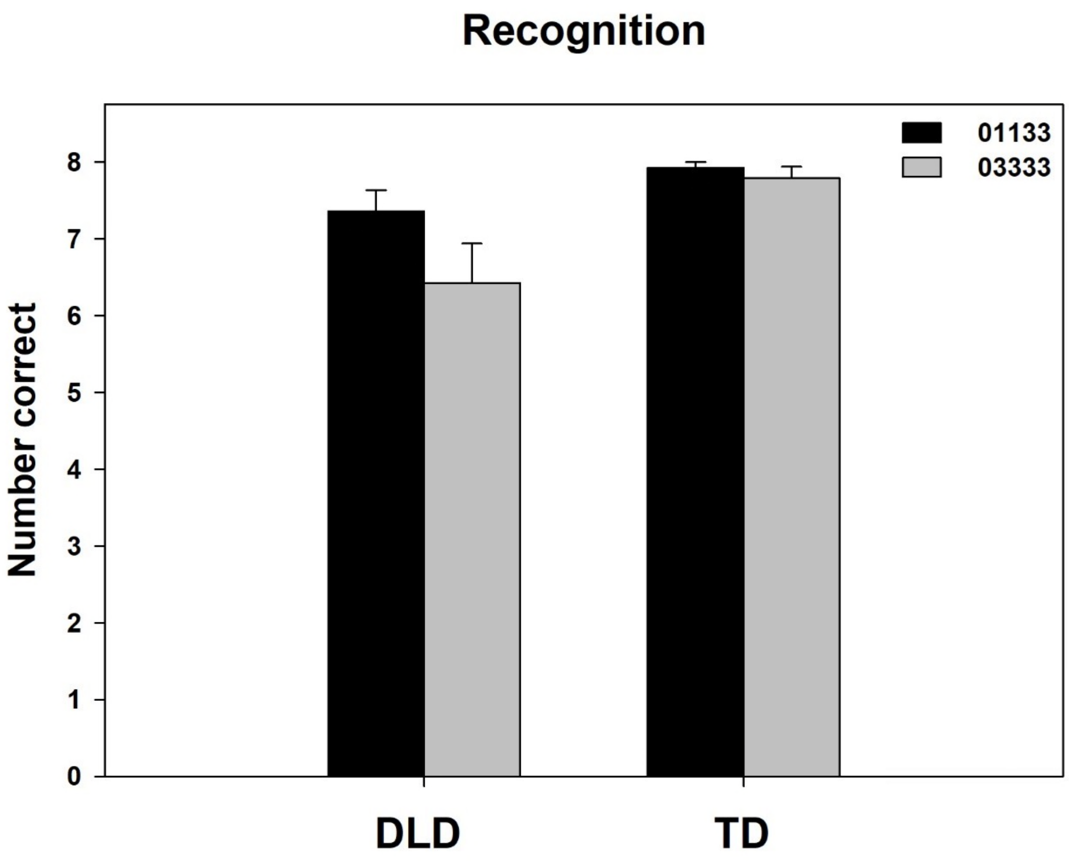

The participant group analyses of the recognition data paralleled the analyses of the data for word form recall. The children with TD had numerically higher scores than the children with DLD. However, the PPVT-5 scores proved relevant—their use as a covariate reduced the effect size for participant group and the effect was no longer different.
